# Bedside estimates of dead space using end-tidal CO_2_ are independently associated with mortality in ARDS

**DOI:** 10.1186/s13054-021-03751-x

**Published:** 2021-09-15

**Authors:** Paola Lecompte-Osorio, Steven D. Pearson, Cole H. Pieroni, Matthew R. Stutz, Anne S. Pohlman, Julie Lin, Jesse B. Hall, Yu M. Htwe, Patrick G. Belvitch, Steven M. Dudek, Krysta Wolfe, Bhakti K. Patel, John P. Kress

**Affiliations:** 1 Section of Pulmonology and Critical Care, University of Chicago, 5841 South Maryland Ave. MC 6026, Chicago, IL 60637 USA; 2 University of Michigan, Ann Arbor, MI USA; 3 Department of Pulmonary Medicine, University of Texas MD Anderson Cancer Center, Houston, TX USA; 4 Division of Pulmonary, Critical Care, Sleep and Allergy, University of Illinois Chicago, Chicago, USA

**Keywords:** ARDS, Mortality, Blood gas analysis, End-tidal CO_2_

## Abstract

**Purpose:**

In acute respiratory distress syndrome (ARDS), dead space fraction has been independently associated with mortality. We hypothesized that early measurement of the difference between arterial and end-tidal CO_2_ (arterial-ET difference), a surrogate for dead space fraction, would predict mortality in mechanically ventilated patients with ARDS.

**Methods:**

We performed two separate exploratory analyses. We first used publicly available databases from the ALTA, EDEN, and OMEGA ARDS Network trials (*N* = 124) as a derivation cohort to test our hypothesis. We then performed a separate retrospective analysis of patients with ARDS using University of Chicago patients (*N* = 302) as a validation cohort.

**Results:**

The ARDS Network derivation cohort demonstrated arterial-ET difference, vasopressor requirement, age, and APACHE III to be associated with mortality by univariable analysis. By multivariable analysis, only the arterial-ET difference remained significant (*P* = 0.047). In a separate analysis, the modified Enghoff equation ((P_a_CO_2_–P_ET_CO_2_)/P_a_CO_2_) was used in place of the arterial-ET difference and did not alter the results. The University of Chicago cohort found arterial-ET difference, age, ventilator mode, vasopressor requirement, and APACHE II to be associated with mortality in a univariate analysis. By multivariable analysis, the arterial-ET difference continued to be predictive of mortality (*P* = 0.031). In the validation cohort, substitution of the arterial-ET difference for the modified Enghoff equation showed similar results.

**Conclusion:**

Arterial to end-tidal CO_2_ (ETCO_2_) difference is an independent predictor of mortality in patients with ARDS.

## Introduction

Acute Respiratory Distress Syndrome (ARDS) is an acute inflammatory process which leads to protein-rich, non-hydrostatic pulmonary edema with reduced lung compliance, refractory hypoxemia, and impaired ability to eliminate carbon dioxide [1]. This inefficiency in CO_2_ elimination is caused by increased physiologic dead space as microvascular injury reduces perfusion of ventilated alveoli. Despite advances in the understanding of the pathophysiology in ARDS and identification of improved strategies that prevent further ventilator-induced lung injury (VILI) [2], mortality in ARDS remains high [1].

Given the high morbidity and mortality of ARDS, it is important to identify reliable prognostic indicators, not only to predict outcomes in individual patients, but also to stratify patients for clinical trials and escalation of supportive measures. In addition to patient characteristics such as age and severity of illness, various physiologic parameters associated with ARDS have been identified to stratify disease severity and outcomes. These physiologic parameters include driving pressure [3], PaO_2_:FiO_2_ ratio, and physiologic dead space [4]. In a prior study by Nuckton et al. [5], the dead-space fraction (Vd/Vt) was measured early in patients with ARDS and was independently correlated with survival. These investigators noted that mean dead-space fraction was markedly elevated (0.58 ± 0.09) early in the course of ARDS and was higher among patients who died than those who survived (0.63 ± 0.10 vs 0.54 ± 0.09; *P* < 0.001).

Unfortunately, measuring lung dead-space at the bedside is not easy. It requires collection of expired gas into a reservoir bag for several minutes and is not routinely employed outside research study circumstances. In contrast, arterial CO_2_ and end-tidal CO_2_ are both measured frequently in critically ill, mechanically ventilated patients. Continuous end-tidal CO_2_ monitoring provides insight into three main functions: metabolism, circulation, and ventilation [6, 7]. The pulmonary pathophysiology of ARDS includes increased dead space fraction in addition to shunt [8, 9]. There are several mechanisms by which increased dead space may occur in ARDS. These include alveolar capillary injury, in situ microvascular thrombosis, and small airways and/or alveolar epithelial injury with V/Q mismatch. Additionally, reduction in right ventricular function may lead to higher dead space by an increase in West zone I and II perfusion [10].

The arterial to end-tidal CO_2_ difference should follow the directional change of the dead space fraction. Accordingly, we hypothesized that the arterial-ET difference would predict mortality in mechanically ventilated patients with ARDS and sought to test this hypothesis with an exploratory analysis in a cohort from the ARDS Network public database (https://biolincc.nhlbi.nih.gov). Following the exploratory analysis, we sought to validate our hypothesis in a cohort of medical ICU patients at the University of Chicago.

## Methods

### Patient cohort

We used the ARDS Network database (https://biolincc.nhlbi.nih.gov) as our derivation cohort. In a review of the eight available ARDS Network databases, three contained both end-tidal CO_2_ and arterial CO_2_ measurements. These three databases (OMEGA [11], ALTA [12], and EDEN [13]) were included in the exploratory analysis.

For our validation cohort, we performed a retrospective analysis of patients with ARDS [4] at the University of Chicago from January 2010 through October 2019. With the assistance of the University of Chicago Center for Research Informatics, we queried our local Epic electronic medical record (Verona, WI) data warehouse using the following criteria: age > 18 years, inpatient or emergency encounters, index encounters, and ICD9/10 codes for ARDS, Acute Hypoxemic Respiratory Failure, Respiratory Failure, Ventilator Support, Hypoxia, and Hypoxemia. The study was approved by the University of Chicago Institutional Review Board (IRB) with a waiver of consent for this de-identified retrospective analysis. We used the STROBE cohort checklist when writing our report [14].

Exclusion criteria included: age < 18, pregnancy, extracorporeal membrane oxygenation (ECMO) during the first 24 h, and those with > 50 pack-year smoking history (to avoid patients with COPD and the inherent pre-existing impact on dead space fraction). We also excluded patients with COPD on pulmonary function tests and/or emphysema on chest high-resolution CT scans based upon a blinded review by a senior pulmonologist (JPK). Lastly, patients without reported end-tidal CO_2_ values within the first 24 h after ARDS diagnosis were excluded.

### Measurements of the arterial-ET difference

Because both derivation and validation cohorts were retrospective analyses, simultaneous arterial and end-tidal CO_2_ measurements could not be collected by protocol. We recorded arterial blood gases measured during the first 24 h after the diagnosis of ARDS was established. In order to be included in the analyses, end-tidal CO_2_ must have been recorded within 1 h of the arterial blood gas with no ventilator changes between arterial and end tidal CO_2_ measurements. For all analyses, we used the arterial CO_2_ and end-tidal CO_2_ measured on day one of ARDS diagnosis only. The physiologic dead space was estimated using two methods. First by calculating the difference between arterial and end-title CO_2_ (P_a_CO_2_–P_ET_CO_2_). Second, we substituted end-title CO_2_ for mean exhaled CO_2_ in the Enghoff modification of the Bohr equation. We will refer to this as the simplified Enghoff modification ((P_a_CO_2_–P_ET_CO_2_)/P_a_CO_2_).

### Statistical analysis

The primary outcome variable for both cohorts was in-hospital mortality. Logistic-regression analysis was used to examine multiple variables in order to determine independent association(s) with mortality. Independent variables were chosen on the basis of prior ARDS studies as well as biological plausibility. All variables associated with mortality by univariable analysis (*P* < 0.10) were included in the multivariable models. We began with our derivation analysis using the 124 patient cohort gathered from the ARDS network database. Demographics, comorbidities, physiological characteristics, ventilator parameters, and outcomes were compared using Chi-square test for categorical variables and Mann–Whitney U for continuous variables.

The following independent variables were considered: age, gender, APACHE score, tidal volume per ideal body weight, arterial-ET difference, positive end expiratory pressure (PEEP), and ECMO use during hospitalization. A second model was created where the simplified Enghoff equation ((P_a_CO_2_–P_ET_CO_2_)/P_a_CO_2_) was substituted for the arterial to end-tidal difference. *P* values less than 0.05 were considered statistically significant for all comparisons. All analyses were performed using Stata 16.1 (College Station, TX) and R [15].

## Results

The ARDS Network database yielded a total of 124 patients to serve as the derivation cohort. Our original query from the University of Chicago medical records found 499 patients for our validation cohort. After review of each record, we identified 302 patients who met our a priori inclusion criteria. The most common reasons that patients were excluded were: COPD/emphysema (*N* = 52), the absence of PaCO_2_ and ETCO_2_ data within an hour of each other (*N* = 112), and other (*N* = 33).

Patient characteristics are summarized in Table 1. When comparing clinical characteristics, the University of Chicago cohort was significantly older, had more severe ARDS, and a higher mortality.

**Table 1:** Clinical characteristics of ARDS patients

	Total	ARDSnet	University of Chicago	*P* value comparing ARDSnet and U of Chicago
	N = 426	N = 124	N = 302	
Age	55.5 (42.1–66.6)	51.0 (40.5–63.0)	57.7 (42.4–67.7)	0.007
Gender				
Male	227 (53.3%)	68 (54.8%)	159 (52.6%)	0.68
Female	199 (46.7%)	56 (45.2%)	143 (47.4%)	
Race				< 0.001
White	188 (44.1%)	88 (71.0%)	100 (33.1%)	
Hispanic	21 ( 4.9%)	11 ( 8.9%)	10 ( 3.3%)	
African American	196 (46.0%)	25 (20.2%)	171 (56.6%)	
Asian	9 (2.1%)	0 (0.0%)	9 (3.0%)	
Other	12 (2.8%)	0 (0.0%)	12 (4.0%)	
SOFA	9.0 (7.0–12.0)	10.0 (7.5–13.0)	9.0 (7.0–12.0)	0.085
APACHE II	NA	NA	29 (23–36)	
APACHE III	NA	90 (72–113)*	NA	
PaO_2_:FiO_2_	136.0 (92.8–197.5)	176.6 (134.0–235.8)	120.0 (81.0–178.0)	< 0.001
Tidal volume	420.0 (362.0–460.0)	427.5 (360.0–480.0)	420.0 (370.0–450.0)	0.71
TV ml/kg IBW	6.4 (5.8–7.2)	6.5 (6.0–7.4)	6.3 (5.7–7.1)	0.002
Hospital mortality	199 (46.7%)	26 (21.0%)	173 (57.3%)	< 0.001

Data are presented as median and interquartile range (IQR) for continuous measures and *n* (%) for categorical measures

*PaO*_*2*_*:FiO*_*2*_ Ratio of arterial partial pressure of oxygen to fractional inspired oxygen, *SOFA* sequential organ failure assessment, *APACHE* acute physiology and chronic health evaluation, *IBW* ideal body weight

Gender, tidal volume per ideal body weight, PEEP, and ECMO use during hospitalization were not significant in univariable analyses for either derivation or validation cohorts. Accordingly, these independent variables were not introduced into the multiple logistic-regression models for either analysis.

In the ARDS Network cohort, four variables were associated with mortality by univariable analysis: arterial-ET difference, age, vasopressor requirement, and APACHE III. By multivariable analysis, only the arterial-ET difference remained significant (Table 2). A model substituting the simplified Enghoff equation in place of the arterial-ET difference also demonstrated the estimation of dead space to be the only significant variable (Table 3).

**Table 2:** Multivariable analysis of derivation cohort using arterial-ET difference: ARDS net database

Mortality	Odds ratio	*P* value	[95% conf. interval]	
Arterial-ET difference	1.10	0.047	1.00	1.21
Age	1.01	0.423	0.98	1.04
APACHE III	1.02	0.093	1.00	1.04
Vasopressor requirement	1.30	0.623	0.45	3.84

*Arterial-ET Difference* arterial end-tidal CO_2_ difference, *APACHE* acute physiology and chronic health evaluation

**Table 3:** Multivariable analysis of derivation cohort using simplified Enghoff equation: ARDS net database

Mortality	Odds ratio	*P* value	[95% conf. interval]	
Simplified Enghoff equation	1.05	0.024	1.00	1.10
Age	1.01	0.462	0.98	1.04
APACHE III	1.02	0.136	1.00	1.03
Vasopressor requirement	1.21	0.716	0.41	3.64

*APACHE* acute physiology and chronic health evaluation

In the University of Chicago validation cohort, five variables were associated with mortality by univariable analysis: arterial-ET difference, ventilator mode, vasopressor requirement, age, and APACHE II. By multivariable analysis, age, APACHE, vasopressor requirement, and the arterial-ET difference remained significant (Table 4). Similarly, replacing the arterial-ET difference with the simplified Enghoff equation, these variables, including estimation of deadspace, continued to be significant variables in the model (Table 5).

**Table 4:** Multivariable analysis of validation cohort: University of Chicago

Mortality	Odds ratio	*P* value	[95% conf. interval]	
Arterial-ET difference	1.03	0.031	1.01	1.06
Age	1.02	0.012	1.01	1.04
APACHE II	1.07	< 0.001	1.04	1.10
Vasopressor requirement		0.029		
Ventilator mode*				
Assist control	–	–	–	
APRV	897,712.43	0.992	8.55 × 10^–102^–NA	
Pressure control	8,997,732.21	0.984	6.72 × 10^–49^–NA	
Pressure support	8,997,732.21	0.992	2.08 × 10^−02^–NA	
SIMV	38.97	0.410	0.0193–2.75	

*Arterial-ET Difference* arterial end-tidal CO_2_ difference, *APACHE* acute physiology and chronic health evaluation

**Table 5:** Multivariable analysis of derivation cohort using simplified Enghoff equation: University of Chicago

Mortality	Odds ratio	*P* value	[95% conf. interval]	
Simplified Enghoff equation	1.02	0.003	1.01	1.09
Age	1.02	0.017	1.00	1.04
APACHE III	1.07	< 0.001	1.04	1.10
Vasopressor Requirement	1.81	0.044	1.02	3.22
Ventilator mode*				
Assist control	–	–	–	
APRV	9,517,519.45	0.992	4.8 × 10^–104^–NA	
Pressure control	6,336,025.64	0.988	7.89 × 10^–50^–NA	
Pressure support	1,046,002.43	0.992	6.3 × 10 ^−106^–NA	
SIMV	0.047	0.51	1.01–1.04	

*APACHE* acute physiology and chronic health evaluation

**Fig. 1:**
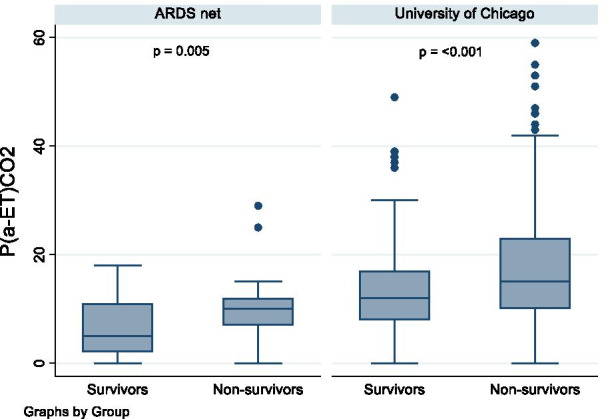
Box plot of arterial end-tidal CO_2_ difference stratified by mortality in the ARDS Net and University of Chicago cohorts

In the ARDS net cohort, the arterial-ET difference was significantly higher in non-survivors (median 10 mmHg, IQR 7–12) compared to those who survived (median 5 mmHg, IQR 2–11) by univariable analysis (*P* < 0.005). The same was seen in the University of Chicago cohort (median 15 mmHg, IQR 10–23 vs median 12 mmHg, IQR 8–17, *P* < 0.001) (Fig. 1). Importantly, the arterial-ET difference was independently associated with an increased risk of death in the multiple regression analysis in both cohorts using either arterial-ET difference or simplified Enghoff estimation of dead space. For every increase of 1 mmHg in the arterial-end tidal CO_2_ difference, the odds of death were 1.10 and 1.03 times higher in the ARDS Network and the University of Chicago cohorts, respectively (Tables 2, 4).

## Discussion

ARDS is a common reason for critical illness and respiratory failure with high mortality [16], making routinely collected measurements that accurately predict outcomes of utmost importance. They may assist in clinical management and prognostication as well as stratification of patients enrolled in clinical trials. Several physiologic parameters available at the bedside have been described to serve these purposes including *P*/*F* ratio [4], oxygenation index [17], driving pressure [3], and dead space [5]. Dead space can be measured by the Bohr method [18], but this requires collection of expired gas and measurement of expired PCO_2_ and arterial PCO_2_. Here, we present that the estimation of dead space by either the arterial-ET difference or the simplified Enghoff modification equation is independently predictive of mortality in critically ill patients with ARDS.

A potential alternative to the direct measurement of dead space is the use of the arterial to end-tidal PCO_2_ difference, which theoretically should be a reliable estimate of dead space. To date, there are limited data supporting this inference. Very early studies by Severinghaus, et al. [19] and others [20, 21] showed that this difference tracked with changes in dead space and the degree of ventilation/perfusion (V/Q) mismatch in *healthy* animal models. Nunn et al. [22] obtained arterial-ET differences in 12 *healthy* anesthetized patients and proposed this measurement as the simplest method of demonstrating the existence of V/Q mismatch. Shetty et al. evaluated 215 patients presenting to the emergency department (ED) and the arterial-ET difference modestly predicted adverse outcomes in patients presenting with suspected sepsis due to *non-respiratory* causes. Those with normal arterial-ET differences were noted to have much lower risk for hospital mortality and prolonged ICU length of stay [23]. Yamanaka et al. [24] studied 17 patients requiring endotracheal intubation and mechanical ventilation using an average of exhaled PCO_2_ at the end of several breaths over a duration of 30 s and found that the difference between arterial and exhaled CO_2_ correlated closely with physiological dead space (*r* = 0.80, *P* < 0.05). Similarly, a prospective study in 106 trauma patients requiring emergency surgery noted that the arterial-ET difference was lower during all phases of surgery in survivors (5.8 ± 4.5 vs. 16.5 ± 14.7 mm Hg) (*P* < 0.001) [25]. In another study that included 412 patients presenting to the ED with shortness of breath, ETCO_2_ was measured with a sampling cannula. A difference > 10 mm Hg was strongly predictive of the need for positive pressure ventilation via face mask or endotracheal tube (AUC 0.91 [95% CI 0.87–0.94]) [7]. In our current study, high estimations of deadspace measured within the first 24 h of ARDS onset were significantly associated with in-hospital mortality. These statistically significant associations persisted with multiple cohorts and when using either the artieral-ET difference or simplified Enghoff equation. Accordingly, we believe that the arterial-ET difference and the simplified Enghoff equation can be useful for early prognostication in ARDS patients in a highly generalizable manner.

Our study has strengths and limitations. Our validation cohort was derived from a single-center analysis. Mortality in the University of Chicago cohort group was significantly higher than the ARDS Network group. This difference is likely explained by the University of Chicago cohort being significantly older and having dramatically worse gas exchange by *P*/*F* ratio. Both derivation and validation cohorts found the arterial-ET difference or simplified Enghoff equation to be an independent predictor of mortality, which speaks to the generalizability of our findings. This is a retrospective study with no precise timing between difference measurements. In order to minimize changes in the difference between arterial and end-tidal CO_2_, we required that the two measurements occurred no more than 1 h apart and with no modifications of ventilator settings. Given that arterial blood-gas and ETCO_2_ measurements are largely dependent on temporal hemodynamic and respiratory factors, our study is limited by potential disparities due to the rapid, time-dependent fluctuations of the measured variables. The use of volumetric capnometry may be a more accurate measurement than ETCO_2_, given that it allows the separation of physiologic dead space from apparent changes in dead space due to shunt and thus can give a more precise indication of physiological mechanism; however, it is often not practical in the ICU setting. Optimization of future investigation of the association between dead space fraction and mortality could include measuring the arterial blood gas and ETCO_2_ at the same time to reduce the risk of hemodynamic changes potentially skewing the data.

## Conclusions

In summary, we identified the arterial-ET difference or the simplified Enghoff equation as independently associated with ARDS mortality. Bedside estimation of dead space early after the diagnosis of ARDS may provide useful prognostic information for ICU care providers.

## Data Availability

The datasets generated and analyzed for the derivation cohort during the current study are available in the ARDS Network Public Database repository, [https://biolincc.nhlbi.nih.gov]. The datasets used and analyzed during the current study for the validation cohort are available from the corresponding author on reasonable request.
